# Using Large Language Models to Detect Depression From User-Generated Diary Text Data as a Novel Approach in Digital Mental Health Screening: Instrument Validation Study

**DOI:** 10.2196/54617

**Published:** 2024-09-18

**Authors:** Daun Shin, Hyoseung Kim, Seunghwan Lee, Younhee Cho, Whanbo Jung

**Affiliations:** 1 Department of Psychiatry Anam Hospital Korea University Seoul Republic of Korea; 2 Doctorpresso Seoul Republic of Korea; 3 VOLTWIN Seoul Republic of Korea; 4 Department of Design Seoul National University Seoul Republic of Korea

**Keywords:** depression, screening, artificial intelligence, digital health technology, text data

## Abstract

**Background:**

Depressive disorders have substantial global implications, leading to various social consequences, including decreased occupational productivity and a high disability burden. Early detection and intervention for clinically significant depression have gained attention; however, the existing depression screening tools, such as the Center for Epidemiologic Studies Depression Scale, have limitations in objectivity and accuracy. Therefore, researchers are identifying objective indicators of depression, including image analysis, blood biomarkers, and ecological momentary assessments (EMAs). Among EMAs, user-generated text data, particularly from diary writing, have emerged as a clinically significant and analyzable source for detecting or diagnosing depression, leveraging advancements in large language models such as ChatGPT.

**Objective:**

We aimed to detect depression based on user-generated diary text through an emotional diary writing app using a large language model (LLM). We aimed to validate the value of the semistructured diary text data as an EMA data source.

**Methods:**

Participants were assessed for depression using the Patient Health Questionnaire and suicide risk was evaluated using the Beck Scale for Suicide Ideation before starting and after completing the 2-week diary writing period. The text data from the daily diaries were also used in the analysis. The performance of leading LLMs, such as ChatGPT with GPT-3.5 and GPT-4, was assessed with and without GPT-3.5 fine-tuning on the training data set. The model performance comparison involved the use of chain-of-thought and zero-shot prompting to analyze the text structure and content.

**Results:**

We used 428 diaries from 91 participants; GPT-3.5 fine-tuning demonstrated superior performance in depression detection, achieving an accuracy of 0.902 and a specificity of 0.955. However, the balanced accuracy was the highest (0.844) for GPT-3.5 without fine-tuning and prompt techniques; it displayed a recall of 0.929.

**Conclusions:**

Both GPT-3.5 and GPT-4.0 demonstrated relatively reasonable performance in recognizing the risk of depression based on diaries. Our findings highlight the potential clinical usefulness of user-generated text data for detecting depression. In addition to measurable indicators, such as step count and physical activity, future research should increasingly emphasize qualitative digital expression.

## Introduction

Depressive disorders are globally prevalent mental health conditions that significantly impact social and occupational functioning [1-3]. Major depressive disorder (MDD) and dysthymia are particularly noteworthy, as together, they were the second leading cause of years lived with disability in 2010, with MDD and dysthymia contributing 8.2% and 1.4%, respectively. The global incidence of depression increased from 172 million in 1990 to 258 million in 2017, reflecting a 49.86% rise, and the associated burden increased by 37.5% from 1990 to 2010 [4,5]. Moreover, depression is a key contributor to increased mortality risk, as evidenced by a meta-analysis indicating a hierarchy in the lifetime prevalence of suicide among patients with affective disorders [6,7]. Early detection and intervention for clinically significant depression have garnered increased attention. Prior to the 2000s, there was insufficient evidence supporting the use of screening tools. However, the United States Preventive Services Task Force revised its stance in June 2002, recommending that physicians screen for MDD [8].

Depressive symptoms are typically evaluated through self-reports or clinical assessment, with notable assessment methods including the Center for Epidemiologic Studies Depression (CES-D) scale, Patient Health Questionnaire–9 (PHQ-9), and Beck Depression Inventory [9-13]. However, these methods have limitations. Patients may underreport symptoms, and there can be discrepancies between subjective reports and objective severity [14,15]. Additionally, individuals often seek initial treatment from general practitioners rather than psychiatry specialists, partly due to stigma and lack of awareness, further complicating accurate assessment [16].

To address these limitations, researchers are exploring various biomarkers, genetic markers, and ecological momentary assessments (EMAs) for more objective and accurate screening [17-19]. EMAs involve real-time data collection, either actively by user input or passively through sensors on wearable devices [20]. Quantitative data such as exercise levels, step counts, and sleep cycles are relatively straightforward to collect and analyze in relation to depression scales [21]. However, analyzing qualitative data, such as user-generated text, presents a more complex challenge.

User-generated text data hold significant clinical potential. Advances in artificial intelligence (AI), particularly in natural language processing (NLP), have enabled sophisticated analysis of such data [22,23]. Large language models (LLMs) like ChatGPT (OpenAI) have facilitated various medical applications, including in psychiatry [24,25]. These models can analyze language used by patients in everyday contexts, such as social media posts, speech, or writing, to detect markers associated with depression [26,27]. By examining linguistic patterns, NLP and LLMs can predict depression risk without relying on traditional survey participation [28].

Despite their potential, much of the text data used in depression research has been sourced from electronic medical records or social media, which may not fully represent natural language use [29]. Daily writing is a universal human activity with therapeutic benefits, including improvements in depression and nonsuicidal self-injury [30-34]. Diary writing supports patient introspection, growth, and communication with therapists, making it a promising EMA data source.

In this study, we aimed to develop an algorithm for depression screening based on user-generated diary text data. Using a daily writing app for emotions, we collected semistructured diary texts and analyzed them with an LLM. Our goal was to validate the clinical utility of these texts as an EMA data source for identifying depression risk.

By leveraging the therapeutic and diagnostic potential of daily writing, combined with the analytical power of LLMs, we seek to contribute to the field of digital mental health. Our approach addresses the limitations of traditional screening tools and highlights the importance of qualitative data in mental health assessment and intervention.

## Methods

### App Process and Diary Log

**Figure 1:**
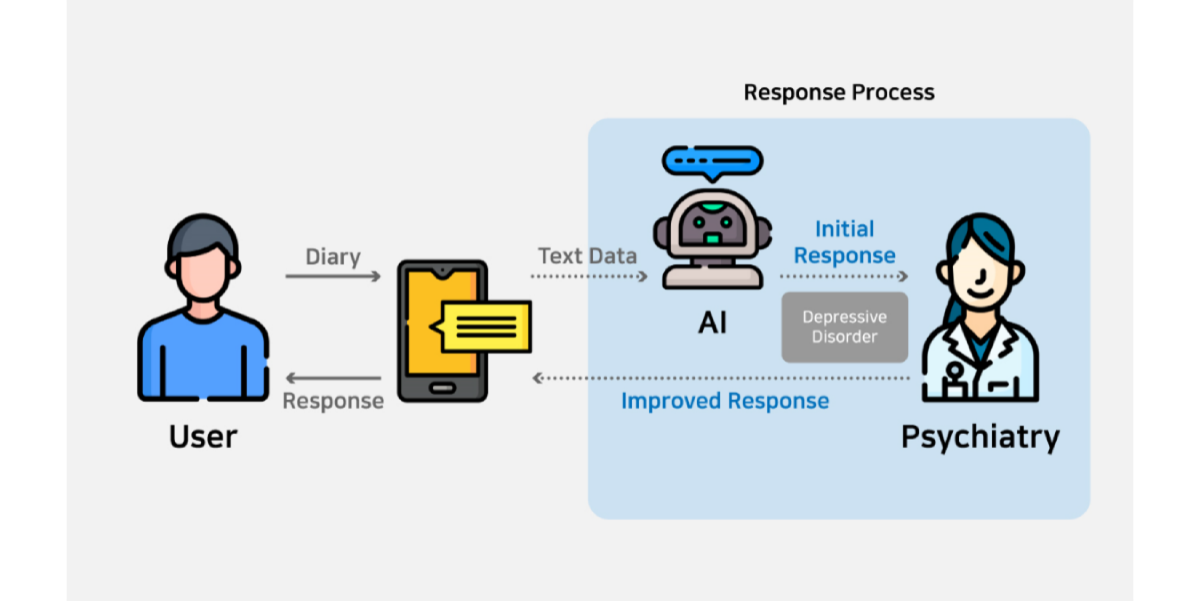
Application process. AI: artificial intelligence.

The text data were obtained through an app named Mind Station. This app prompts users to write daily emotional diaries. Based on diary data, an AI system assesses the risk of depression and provides this information to mental health professionals. In addition, it uses generative AI to create responses to emotional diaries. A psychiatric clinician reviews these responses to modify and enhance them in a cognitive-behavioral or supportive psychotherapeutic manner, then provides replies to the user’s diary (Figure 1).

The daily diary log was divided into 4 paragraphs, beginning with a description of the events that occurred that day, followed by reflections on these events and the resulting emotions, and concluding with a free-form diary entry. For each paragraph, the limit was set to a maximum of 500 characters to ensure brevity. Based on the written diaries, a specialist in mental health and medicine provided individualized emotional support. This step involved encouraging exploration, rectifying distorted cognition, guiding antidepressive activities in daily life, and recommending medical assistance (if necessary). Our objective was to enhance mental health through numerous responses tailored to individual needs.

Furthermore, this app was not developed for research purposes but rather as a form of mental self-care. However, we had plans to collect various digital phenotype data through the Mind Station app and to build algorithms for diagnosing and treating depression by analyzing text data. Therefore, users of the app were allowed to write diaries and receive responses from clinical psychiatrists only after agreeing that their anonymized data could be used for future research purposes.

### Recruitment

We recruited 91 participants from October 1, 2022, to April 30, 2023. Participants were recruited through promotion within internet communities for beta testing of the app. These promotions informed individuals about an app for writing diaries to care for their mental health where they could receive free responses from clinical psychiatrists. Additionally, they were notified that their anonymized data might be used for future research purposes. The participants received a reward of approximately ₩30,000 (US $22) for completing diaries for at least 1 week, writing daily, and submitting self-reported questionnaires about their mood. Furthermore, they voluntarily agreed to receive information on data collection and use before using the app. In addition, rewards were only disbursed after the completion of the week-long app experience and the submission of feedback regarding technical errors or diary responses encountered in the app. This was done to reduce the likelihood of participants writing diaries arbitrarily. Researchers should collect well-established risk factors, such as sex, family history, and employment status, for depression screening. However, an increase in the amount of input information can lead to lower compliance, potentially diminishing its role as a screening tool. Therefore, we performed an analysis based solely on text data from diaries written by users and from self-reported questionnaires. Diaries that were filled only with words lacking substantive content, such as “lol,” or solely with names of other individuals who were present at a particular location, were excluded from the analysis. This study aimed to retrospectively analyze the collected data.

### Ethical Considerations

This study adhered to the ethical principles outlined in the Helsinki Declaration. Approval was obtained from the Institutional Review Board of Korea University Anam Hospital (2023AN0379). Each participant received approximately ₩30,000 (US $22) for completing diaries as long as they fulfilled the criteria described above. Data were anonymized. The participants were informed and consented to the use of their data for research purposes upon accessing the app. As this study involved retrospective analysis of the data, no written informed consent was obtained.

### Depression Assessment Scale, Diary Classification, and Statistics

All participants were evaluated for depressive symptoms using the PHQ-9 and for potential crises in suicide situations using the Beck Scale for Suicide Ideation (BSS). The PHQ-9 consists of 9 questions related to mood, sleep, and other factors, with scores ranging from 0 to 3 for each question. The total score ranges from 0 to 27 [35]. The optimal cutoff score for depression screening is 10, with scores ≥11 indicating a risk of depression [36]. The BSS is a self-reported questionnaire for assessing suicide risk, and its potential as a screening tool in emergency rooms and inpatient settings has been validated [37-39]. These scores are well established in clinical research, with the PHQ-9 widely recognized for its validity and reliability in assessing depressive symptoms.

The classification of diaries as depressive was based on a combination of the validated PHQ-9, the BSS, and the clinical psychiatrist’s review. The primary criterion for determining a “true” classification was the PHQ-9 and BSS scores. A participant was classified as “depressed” if their PHQ-9 score was 10 or higher or if their BSS score was 8 or higher at the closest point to the diary-writing day. For diaries without available PHQ-9 and BSS scores, a psychiatrist reviewed the content. Diaries were classified as depressive if they contained direct expressions of depression, such as “I want to die” or “I am depressed,” or if they had clinically recognizable signs of depression, like “I am very stressed.” Additionally, if a participant’s PHQ-9 score was 10 or higher at the time of writing the first diary entry, but subsequent diaries (4-5 days later) included statements indicating an improved mood, such as “I feel better” or “Today was a good day,” the psychiatrist reviewed these diaries and classified them as not exhibiting signs of depression. This dual approach of using validated depression scales and clinician review ensured a comprehensive and accurate classification of the diaries, balancing quantitative and qualitative assessments.

The statistical analysis method used for comparing depression and suicide scores before and after diary writing was a 2-tailed Student *t* test. Statistical analyses were conducted using SPSS Statistics (version 24.0; IBM Corp).

### AI Technology and Data Preprocessing

**Figure 2:**
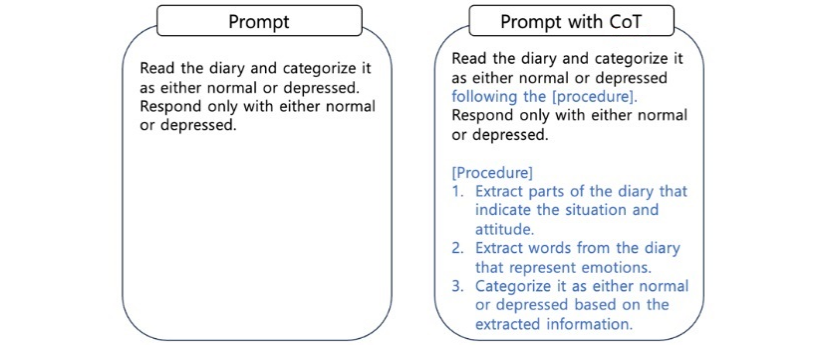
Chain-of-thought (CoT) prompting. Differences with standard prompting are shown in blue.

LLMs have achieved remarkable success in the field of NLP. Initially, we applied the most popular LLMs, such as ChatGPT with GPT-3.5 and GPT-4 [40], to our data set without fine-tuning. In addition, we used a GPT-3.5 fine-tuning model with our training data set. Furthermore, we compared the performance of each model by applying chain-of-thought (CoT) and zero-shot prompting (Figure 2) [41,42]. CoT prompting, zero-shot prompting, and few-shot prompting were defined as follows in this study: CoT prompting is a method used in NLP to guide the model in generating intermediate reasoning steps leading to the final answer. Instead of directly producing an answer, the model generates a sequence of logical thoughts or steps connecting the input to the output. Zero-shot prompting refers to a scenario where an LLM is presented with a task without any prior specific examples or training on that task. The model relies on its preexisting knowledge and understanding to generate an appropriate response based on the provided prompt. Few-shot prompting is a technique in NLP where a model is given a small number of examples (usually ranging from one to a few dozen) of a task to learn from before being asked to perform the task itself. These examples aid the model in better understanding the task and improving its performance. Using the CoT approach, we structured the text and analyzed the words, expressions, and emotions used to classify it. Furthermore, we incorporated additional methods to compare the similarity of familiar texts, such as a compression algorithm based on the GNU Zip (Gzip) algorithm and k-nearest clustering. This step helped us compare the differences between the LLMs and conventional algorithms [43,44]. We adopted stratified 5-fold cross-validation to classify diaries while preserving the percentage of the ratio of labels. We divided the entire data set into training and test data sets at a ratio of 8:2.

In this study, we defined and calculated key metrics to evaluate the performance of our classification models. Recall, formerly known as sensitivity, is the proportion of actual positives correctly identified by the model. It is calculated by dividing the number of true positive predictions by the sum of true positive and false negative predictions. This metric helps in understanding how well the model can identify positive instances. Precision is the proportion of true positive predictions among the total positive predictions. It is calculated by dividing the number of true positive predictions by the sum of true positive and false positive predictions. Precision indicates the accuracy of the model’s positive predictions. Specificity is the proportion of actual negatives correctly identified by the model. It is calculated by dividing the number of true negative predictions by the sum of true negative and false positive predictions. This metric is crucial for understanding the model’s ability to correctly identify negative instances. Accuracy is the overall proportion of correct predictions, including both true positives and true negatives. It is calculated by dividing the sum of true positive and true negative predictions by the total number of predictions, which includes true positives, false positives, false negatives, and true negatives. Accuracy provides a general measure of how well the model performs across all classes [45].

## Results

We collected 428 diaries from 91 participants, with an average of 4.7 (SD 4.44; median 7.0) diaries authored by each user. Among the participants, 34 consented to disclosing their sex and 31 consented to disclosing their age. At baseline, 85 participants responded to the PHQ-9, and at the end of the 2-week period, 34 participants responded. Similarly, 85 participants responded to the baseline BSS, and 30 participants responded at the end of the 2-week period. Among the respondents, 81% (25/31) were women, and 19% (6/31) were men. Regarding age, 87% (27/31) of the total participants were aged 20-39 years, while 13% (4/31) were aged 40-49 years. Every participant underwent an initial assessment using the PHQ-9 and BSS while writing their diaries. At baseline, 85 participants completed the PHQ-9. Their average score was 7.353 (SD 6.849), and 26 participants had scores of 10 or higher. Additionally, 85 participants completed the baseline BSS, and 30 participants completed it again at the end of the 2-week period. The average baseline BSS score was 4.200 (SD 5.708). Among the participants, 2 had PHQ-9 scores of 10 or lower and BSS scores of 8 or higher. As described in the Methods section, even if the initial PHQ-9 and BSS scores exceeded the cutoff points, diaries written 4-5 days later that included direct expressions of improved mood, such as “I feel better,” were classified as nondepressive diaries based on the psychiatrist’s judgment. Therefore, the total number of depressive diaries was 73. Furthermore, the PHQ-9 and BSS scores when terminating use of the app were lower than the scores at the beginning of use (PHQ-9: *P*=.32; BSS: *P*=.40) (Table 1).

**Figure 3:**
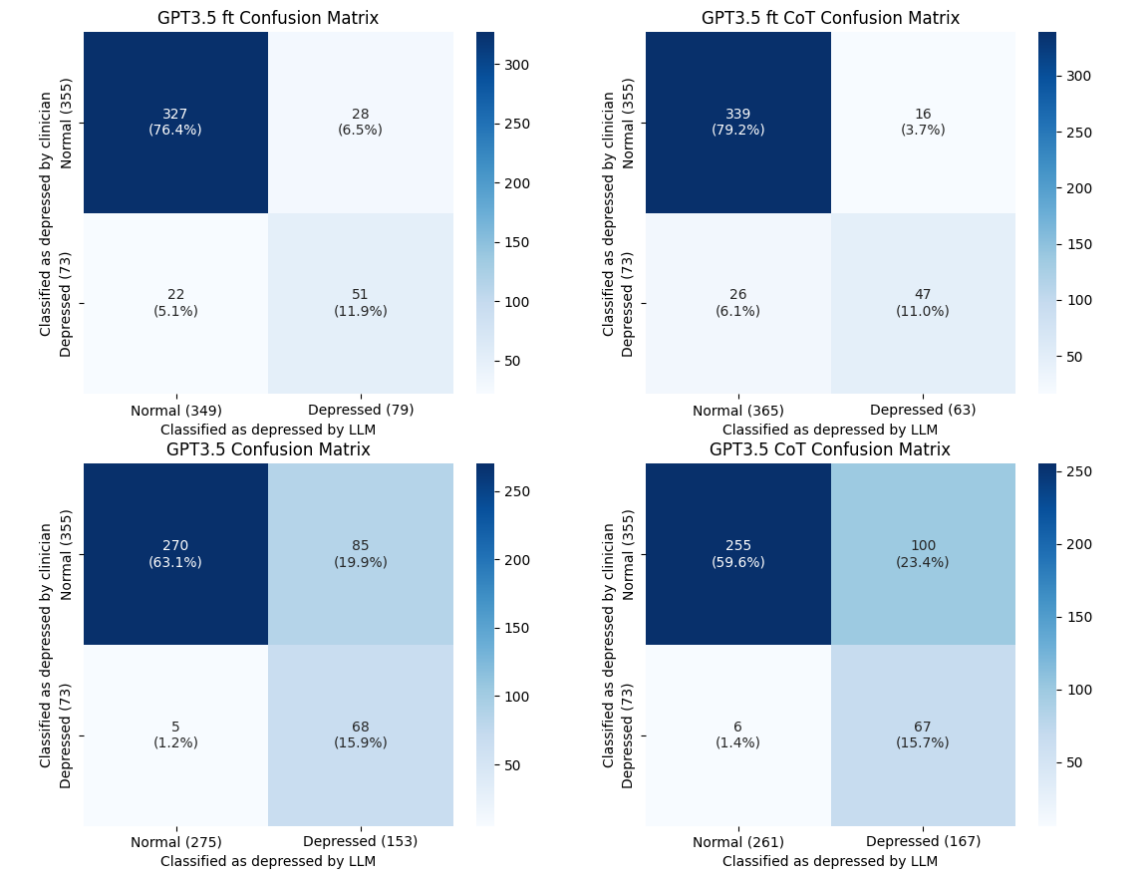
Confusion matrix for GPT 3.5 models. CoT: chain of thought; LLM: large language model.

**Figure 4:**
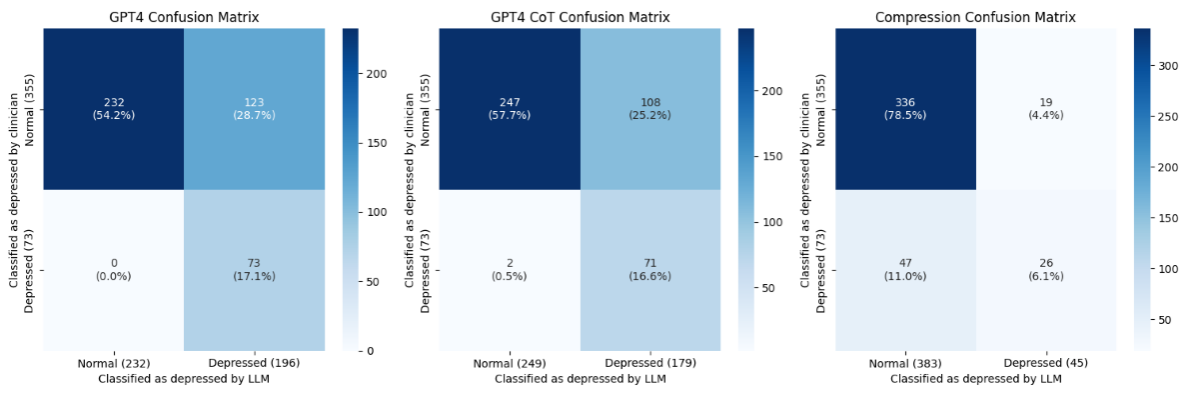
Confusion matrix for other artificial intelligence models. CoT: chain of thought.

Balanced accuracy was calculated as the average of the recall and specificity divided by 2, representing the accuracy for each class. This step is particularly useful for evaluating classes in unbalanced data sets. We used the *F*_1_-score, accuracy, precision, recall, and specificity as evaluation metrics. GPT-3.5 fine-tuning demonstrated the best performance, with an accuracy of 0.902, *F*_1_-score of 0.685, precision of 0.759, and specificity of 0.955. The best fold exhibited higher performance, with an accuracy of 0.942 and an *F*_1_-score of 0.8. However, the recall for GPT-3.5 fine-tuning was moderate, at 0.643. By contrast, GPT-4 demonstrated the best performance, with a recall of 0.972. Nevertheless, GPT-4 lagged behind GPT-3.5 in fine-tuning regarding accuracy (0.743) and *F*_1_-score (0.57) (Table 2). Figures 3 and 4 depict the combined confusion matrix for all folds of each model.

**Table 1:** Characteristics of participants.

Characteristics		Values
**Sex of participants, n** **(%)**		
	Male	6 (19)
	Female	25 (81)
**Age group of participants (years), n** **(%)**		
	20-39	27 (87)
	40-49	4 (13)
**Patient Health Questionnaire–9 score** **, mean** **(SD)**		
	Baseline (n=85)	7.353 (6.849)
	End (n=34)	5.735 (6.336)
**Beck Scale for Suicide Ideation score** **, mean** **(SD)**		
	Baseline (n=85)	4.200 (5.708)
	End (n=30)	2.967 (5.436)

**Table 2:** Performance of each algorithm with and without chain-of-thought (CoT) prompting. Italics indicate high performance.

Algorithm			Accuracy		Balanced accuracy		*F*_1_-score		Precision		Recall		Specificity
**Gpt3.5_ft**													
	Average	*0.883*		*0.808^a^*		0.670		0.653		0.695		*0.921^b^*	
	Maximum	0.930		0.853		0.786		0.846		0.733		0.972	
**Gpt3.5_ft_CoT**													
	Average	*0.902^b^*		0.799		0.685		0.759		0.643		*0.955^c^*	
	Maximum	0.942		0.833		0.800		1.000		0.667		1.000	
**Gpt3.5**													
	Average	0.789		*0.844^a^*		0.607		0.453		*0.929^b^*		0.761	
	Maximum	0.860		0.915		0.714		0.556		1.000		0.831	
**Gpt3.5_CoT**													
	Average	0.752		*0.818^a^*		0.560		0.405		*0.917^b^*		0.718	
	Maximum	0.802		0.828		0.605		0.464		0.867		0.789	
**Gpt4**													
	Average	0.713		*0.827^a^*		0.546		0.378		*1.000^c^*		0.653	
	Maximum	0.791		0.873		0.625		0.455		1.000		0.746	
**Gpt4_CoT**													
	Average	0.743		*0.834^a^*		0.570		0.406		*0.972^c^*		0.696	
	Maximum	0.826		0.894		0.667		0.500		1.000		0.789	
**Compression**													
	Average	*0.846^a^*		0.651		0.431		0.584		0.356		*0.947^b^*	
	Maximum	0.919		0.819		0.741		0.833		0.667		0.972	

^a^Performance >0.80.

^b^Performance >0.90.

^c^Performance >0.95.

## Discussion

### Principal Findings

In this study, log data comprising 428 daily diaries were collected from 91 participants. We predicted the risk of depression based solely on text data from the diaries, excluding other clinical data. Approximately 80% of the users were women, and the majority were aged 20-39 years. The participants were prompted to write diaries about their moods and daily experiences. The average initial PHQ-9 score was 5.719, indicating most users did not exhibit a significant risk of depressive symptoms. The GPT-3.5 model, combined with CoT prompting, achieved an accuracy of 0.902 and a specificity of 0.955 in classifying high-risk cases of depression based on diary text data. GPT-4.0 showed superior recall (1.000) compared to the GPT-4.0 CoT model (0.972), although both models demonstrated similar balanced accuracy. The high imbalance in the data set, with 82.9% (355/428) nondepressive and 17.1% (73/428) depressive diaries, underscores the importance of using balanced accuracy as a metric.

The analysis reveals that while the GPT-3.5 fine-tuned model had high accuracy and specificity, suggesting effectiveness in predicting false outcomes, its relatively low balanced accuracy implies potential overfitting during the fine-tuning process and inadequate prediction of true outcomes. On the other hand, GPT-4 achieved perfect recall but may have overlooked errors in false predictions due to its focus on distinguishing between normal and abnormal data. This raises concerns about false predictions with the GPT-4 model. Despite GPT-4’s lower performance compared to GPT-3.5, both models had balanced accuracy metrics that remained similar. The similarity in performance can be attributed to factors such as the sentiment task nature of the query and the reinforcement of ethical filters in GPT-4. These findings align with the existing literature and highlight the need for further investigation into AI model performance and ethical considerations in sentiment analysis tasks.

Language, particularly text data, excluding voice, is used by individuals and reflects their values and emotions. It can be utilized for EMA. However, various methodological limitations make it challenging to use EMA for depression screening. Previous research on diagnosing or detecting depressive symptoms using text has been based on data from various sources, including time-based narratives, suicide notes, and publicly available social media content. These studies aimed to detect depression not by analyzing the text itself but by extracting various indicators, such as the counts of positive and negative words. These indicators were used as proxies for depression detection [46-48]. Furthermore, social media posts and poems often contain abbreviations or words that are not typically used in everyday, natural-language conversations. Thus, using such data as effective digital biomarkers poses limitations because they may not represent real-life communication accurately. With the evolution of various text analysis technologies, such as NLP, the scope of text used for screening and diagnosing depression has expanded to include electronic medical records [49,50]. However, the language used in electronic medical records is often influenced by the patient’s clinical judgment, which can result in an altered representation. Consequently, text-based depression screening based on electronic medical record data has limitations and poor generalizability.

Fine-tuning is the process of optimizing a pretrained model through additional training for a specific domain or task. Concentrated learning of data related to a specific domain or task can improve performance while conducting tasks related to that domain. CoT is a simple and popular prompting algorithm for improving the performance of language models without fine-tuning. Our results demonstrate the performance of GPT-3.5 and GPT-4 on classification tasks and their improvement upon techniques for additional prompting and fine-tuning. The compression algorithm Gzip is an easy and lightweight nonparametric alternative to neural networks used for text classification. Gzip demonstrates good performance even in out-of-distribution data sets. We determined the results that would emerge from using existing technology instead of deep learning.

Mood diaries exert therapeutic effects. Our results suggest lower depression and suicide scores after completing diary writing than scores after commencing; nonetheless, the difference was statistically insignificant. This finding is partly attributable to the insufficient sample size to prove statistical power. Moreover, the study was conducted in the general population, and few participants demonstrated significantly higher levels of depressive symptoms. The therapeutic effects of diary writing on depression should be confirmed through large-scale studies with a larger sample size.

This study confirmed that emotion diaries among the general population can be used to screen for depression at a level similar to that of passive EMA. The area under the receiver operating characteristic curve of depression through passive sensing depends on the data used; however, the accuracy ranges from approximately 60% to 90% [51]. Text-based depression prediction has a lower accuracy (approximately 0.632); nonetheless, the accuracy of depression diagnosis increases upon using text data from medical interviews along with other data, such as vocal features [52]. Accuracy increases upon analyzing a daily mood diary rather than predicting depression and suicidal thoughts through EMA data obtained through a wearable device [53]. This study was conducted in a relatively large population; however, it has the disadvantage of poor generalizability because the targets were medical interns. A previous study suggested that journaling by older adults can help detect depression, and through this study, we confirmed that depression detection based on journaling can be extended to other age groups [54].

The findings of this research have important clinical implications. If depression could be diagnosed based on daily diaries, it would offer significant clinical benefits. Early detection could identify at-risk individuals, allowing for timely interventions like counseling or therapy to prevent symptom progression. Analyzing diary text data could reveal triggers and patterns, informing personalized therapy approaches such as cognitive behavioral therapy to address negative thought patterns or environmental factors. Regular diary analysis could monitor treatment effectiveness, providing real-time feedback for adjustments and improving patient outcomes. In summary, leveraging daily diaries for depression diagnosis could revolutionize clinical practice by enabling early intervention, personalized therapy, and ongoing treatment monitoring.

### Strengths

The strength of this study is that we confirmed the basis for predicting mild levels of depression. This is because the recruitment of participants was not centered on hospitals. Most text data, excluding social media data, are based on medical records. Therefore, the data are supposedly biased toward individuals with severe depression. An existing depression diagnostic algorithm based on the frequency of negative language demonstrated low accuracy upon application to our data [52]. In addition, the text data are meaningful in that they can be used for simultaneous diagnosis and treatment because writing a diary is already therapeutic. Therefore, they can be a good evaluation tool for determining the severe burden placed on patients from obtaining active EMA data [55]. In addition, we confirmed that prompt engineering alone affected depression detection. Thus, generative AI can facilitate medical judgment, and prompt technology is important.

### Limitations

This study has several limitations. First, the complete exclusion of other clinical data, such as sex, age, and occupation, serves as both an advantage and a disadvantage. The advantage is that it confirms the diagnostic accuracy of pure text data; however, the sample size was small, and the predominant users were women aged 20-39 years. Therefore, the data are possibly biased. Second, we used generative AI; thus, we could not confirm the clinical characteristics recorded in the depression diary. Existing studies have demonstrated clinical implications, such as the use of *I*-centered words in suicide diaries and the frequent use of negative language in social media about depression [46,48]. In addition, the small sample size warrants large-scale research to diagnose depression using written diaries and the clinical characteristics of patients with depression. Third, text data may have a high possibility of being used in combination with other biomarkers; however, such data were not collected in this study. Therefore, further research is required on various types of EMA data that can be used in algorithms to differentiate depression in the general population.

### Conclusions

We investigated whether an LLM can detect depression based on user-generated emotional diaries. Using a data set comprising 428 diaries from 91 users, the accuracy of predicting depression increased upon applying the CoT prompt technique to both GPT-3.5 and GPT-4.0. GPT-3.5 generated the highest average accuracy (0.902). However, without fine-tuning or prompting techniques, GPT-3.5 exhibited the highest balanced accuracy of 0.844 and recall of 0.929. Despite decreased depressive scores after the participants began writing their diaries, this change was statistically insignificant. Therefore, additional research is warranted to explore the potential antidepressant effects of diary writing. Based on these findings, we identified the potential clinical utility of voluntarily generated text data for detecting depression. Beyond quantifiable indicators, such as step count, daily physical activity, and sleep duration, we consider that there will be a continued and increased focus on qualitative digital expressions in research.

## Abbreviations

AI: artificial intelligence
BSS: Beck Scale for Suicide Ideation
CES-D: Center For Epidemiologic Studies Depression
CoT: chain-of-thought
EMA: ecological momentary assessment
Gzip: GNU Zip
LLM: large language model
MDD: major depressive disorder
NLP: natural language processing
PHQ-9: Patient Health Questionnaire–9
