# Correction: Using Large Language Models to Detect Depression From User-Generated Diary Text Data as a Novel Approach in Digital Mental Health Screening: Instrument Validation Study

**DOI:** 10.2196/79198

**Published:** 2025-07-08

**Authors:** Daun Shin, Hyoseung Kim, Seunghwan Lee, Younhee Cho, Whanbo Jung

**Affiliations:** 1 Department of Psychiatry Anam Hospital Korea University Seoul Republic of Korea; 2 Doctorpresso Seoul Republic of Korea; 3 VOLTWIN Seoul Republic of Korea; 4 Department of Design Seoul National University Seoul Republic of Korea

In “Using Large Language Models to Detect Depression From User-Generated Diary Text Data as a Novel Approach in Digital Mental Health Screening: Instrument Validation Study” (J Med Internet Res 2024;26:e54617) the authors noted one error.

The grant number has been corrected from:

This work was also supported by a grant from the Korea Health Technology R&D Project through the Korea Health Industry Development Institute funded by the Ministry of Health & Welfare, Republic of Korea (grant HI23C1234).

to:

This work was also supported by a grant from the Korea Health Technology R&D Project through the Korea Health Industry Development Institute funded by the Ministry of Health & Welfare, Republic of Korea (grant HI23C0035).

The correction will appear in the online version of the paper on the JMIR Publications website, together with the publication of this correction notice. Because this was made after submission to PubMed, PubMed Central, and other full-text repositories, the corrected article has also been resubmitted to those repositories.

